# Morphohistometric analysis of the effects of *Coriandrum sativum* on cortical and cerebellar neurotoxicity

**DOI:** 10.22038/AJP.2021.18107

**Published:** 2021

**Authors:** Hesham N. Mustafa

**Affiliations:** 1 *Department of Anatomy, Faculty of Medicine, King Abdulaziz University, Jeddah, Saudi Arabia*

**Keywords:** Coriandrum sativum Cerebellum, Toxicity, Chelation, Lead

## Abstract

**Objective:**

Natural compounds can act as metal chelators and oxygen free radical scavengers, which allows them to be used as bioactive antagonists to heavy metals neurotoxicity. The aim of the study to analyze the morphometric effects of *Coriandrum sativum* (*C. sativum*) on lead-induced neurotoxicity.

**Materials and Methods:**

Forty Sprague-Dawley albino rats were divided into four equal groups (ten in each group): control group; coriander group: received aqueous *C. sativum* extracts (600 mg/kg BW for 60 days orally); lead (Pb) group: received a daily dose of lead acetate (Pb) (10 mg/kg BW for 60 days orally); Pb+ coriandrum group: received: aqueous *C. sativum* extract (600 mg/kg BW) prior to 10 mg/kg BW of Pb. The following parameters malondialdehyde (MDA), superoxide dismutase (SOD), catalase (CAT) and glutathione peroxidase (GPx) were measured. Layers thickness and nuclei density were analyzed.

**Results:**

Lead levels in blood and tissues were decreased significantly in the Pb group and those findings were corrected significantly (p=0.001) with *C. sativum* addition. Data exhibited an increase in oxidative stress marker MDA and a decrease in antioxidant enzymes activities (SOD, CAT, and GPx) significantly in the Pb group and those effects were reversed significantly (p=0.001) by *C. sativum* administration. The cerebellar cortex and all layers of the somatosensory cortex thickness and nuclei density were diminished significantly in the Pb group. The morphometrical measurements were corrected significantly (p=0.001) by *C. sativum*.

**Conclusion:**

From the findings of the current study, Pb caused noticeable structural and functional variations in the cerebellar cortex and somatosensory cortex. *C. sativum* corrected these parameters as it possesses chelating and antioxidant potentials.

## Introduction

Lead is considered a highly dangerous toxicant which is determined by its capability to accumulate in the body (Mustafa and Hussein, 2016 ▶). The influence of low-dose lead in the postnatal and prenatal on the nervous system is reflected in previous articles (Mustafa and Hussein, 2016 ▶; Saleh et al., 2019 ▶). There is a direct relationship between blood lead levels and decreased intelligence quotient, neurodegenerative deficits and hearing loss in children (Surkan et al., 2007 ▶). Evidence has shown that lead poisoning can stimulate cellular degradation through production of reactive oxygen species (Mehrandish et al., 2019 ▶). So, studies on the morphometric indices of neurons in different parts of the brain are promising (Mustafa and Hussein, 2016 ▶; Saleh et al., 2019 ▶). 

*Coriandrum sativum* is an annual herb, and its dried seeds and fresh leaves are one of the most important spices in the world especially Mediterranean region of Europe (Al-Rubaye, 2016 ▶). Both cilantro and coriander come from the *C. sativum* plant. Cilantro is the name for the plant's leaves and stem, while coriander is the dried seeds, were found in ancient Egyptian tomb. Furthermore, coriander has culinary value and a wide range of healing properties, through food detoxification and removing toxic mineral residue such as lead and excreting them (Tellez-Lopez et al., 2017 ▶). Study of lead-intoxicated and treated with *Coriandrum sativum* showed encouraging results as chelation and poisoning reduction in animal models (Velaga et al., 2014 ▶).

*C. sativum* leaves have been used as appetizer, dyspeptic, anorexic and antispasmodics. Furthermore, it has antidiabetic, antihyperlipidemic, antimicrobial, antioxidant, and memory enhancing effects (Al-Rubaye, 2016 ▶).

The purpose of the study was to examine the ameliorative role of *C. sativum* on the structural state of cerebral cortex neurons against a low toxic dose of lead.

## Materials and Methods

**Ethical approval**

This study was conducted in strict accordance with the recommendations of the National Institutes of Health's Guide for the Care and Use of Laboratory Animals. The research protocol with animal experimentation was approved by the Scientific Ethics Committee of Faculty of Medicine, King Abdulaziz University (No. 227-34). 

**Materials**

Lead acetate trihydrate [(C_2_H_3_O_2_)2Pb.3H_2_O] (PbAc) was purchased from Millipore Sigma Co. (St. Louis, Missouri, USA). A 0.5% of Pb solution was prepared by dissolving 5 g of PbAc in 1000 ml of distilled acidified water (DIH_2_O); the solution was replaced daily to minimize the presence of lead precipitates. A subsequent amount of 5N HCl was added to lead acetate solution to prevent the precipitation of lead salts (Saleh et al., 2019 ▶).

*C. sativum* seeds were collected from local market. Seeds were ground to a powder and 100 g of the powder was added to 500 ml distilled water; after 24 hr maceration at room temperature, after that the mixture was heated for 30 min at 65°C. The extract was filtered, concentrated by heating over a water bath (65°C) and dried under vacuum with the yield of 5.9% (w/w). The extract was stored at 4°C (Veena et al., 2011).

**Experimental design**

**Animals**

Forty Sprague-Dawley albino rats weighing 190±10 g were obtained from the animal house at the King Abdulaziz University and distributed randomly into four groups (n=10). Rats were kept in metallic cages under lighting (12 hr/12 hr light/dark), humidity (55±10%) and standard temperature (22±2ºC) conditions. They were fed a standard chow diet *ad libitum *with free access to water.

Group 1, control group, received 1 ml distilled water (DH20). Group 2 received aqueous *C. sativum* extract (600 mg/kg BW) (Leena et al., 2011 ▶; Donia, 2019 ▶). Group 3 received 10 mg/kg body weight (BW) of lead acetate (PbAc) (Mustafa and Hussein, 2016 ▶; Saleh et al., 2019 ▶). Group 4 received aqueous *C. sativum* extract (600 mg/kg BW) prior to 10 mg/kg BW of PbAc. Administration of the chemicals was done by oral gavage for a period of 60 days.

**Pb levels in** **blood and** **brain** **tissues**

Left halves of cerebral cortices and cerebella from all groups were digested in concentrated nitric acid (100,441, Suprapur® HNO_3_ 65% w/w, MerckMillipore, Darmstadt, Germany) using a shaking water bath at 60°C for 30 min. After digestion, the solution was diluted (1:5 v/v) with DIH_2_O. Lead levels (Pb) were measured using an atomic absorption spectrophotometer (Perkin-Elmer Model 3030, Hopkintin, MA, USA). Results are reported as µg Pb/dl blood, and Pb levels in brain tissues are reported as µg/g tissue weight (Saleh et al., 2019 ▶).

**Biochemical assays**

Left halves of the cerebral cortices and cerebella from all groups were homogenized (10% w/v) in ice-cold 0.1 M sodium phosphate buffer (pH 7.4). The homogenate was centrifuged twice at 4,000 rpm for 15–20 min at 4°C, and the resultant supernatant was used for estimation of various biochemical parameters. Lipid peroxidation (LPO) was estimated by determining the amount of malondialdehyde (MDA), which is formed by peroxidation of membrane lipids using a thiobarbituric acid-reactive substances (TBARS) (QuantiChrom™ TBARS Assay Kit, DTBA-100, BioAssay Systems, Hayward, CA, USA). The antioxidant enzyme activity in cerebral cortices and cerebella was evaluated by determining superoxide dismutase (SOD) activity using a Superoxide Dismutase Assay Kit (706,002, Cayman Chemical, Ann Arbor, MI, USA), in which a tetrazolium salt is used to detect superoxide radicals generated by xanthine oxidase and hypoxanthine. Catalase (CAT) activity was assayed according to the peroxidatic function of catalase using a Catalase Assay Kit (707,002, Cayman Chemical). Glutathione peroxidase (GPx) activity was assayed by coupling the enzyme procedure with glutathione reductase using a Glutathione Reductase Assay Kit (703,202, Cayman Chemical) (Saleh et al., 2019 ▶).

**Histological preparation**

Right halves of the cerebral cortices and cerebella from all experimental groups were dissected; the somatosensory cortex and the cerebella were removed, and then fixed in 10% neutral buffered formalin. Paraffin sections of 5 µm in thickness were prepared. For each specimen, at least 3 to 5 slides were stained with haematoxylin and eosin (H&E) and Nissl stain (cresyl violet) using standard techniques for general histology examination, and examined using an Olympus BX53 microscope equipped with an Olympus DP73 camera (Olympus, Tokyo, Japan) for the observation of degenerative changes.

**Image capture and quantitative morphometric analysis**

For each stained section, 10 non-overlapping fields were measured for each parameter; a mean value was calculated, and analyzed using Image-Pro Plus v6 (Media Cybernetics Inc., Bethesda, Maryland, USA). ImageJ (National Institute of Health, Bethesda, MD, USA) [version 1.53a] was used to quantify cells and measure the thickness of the layers of the cerebellar cortex and somatosensory zone of the hemispheres, and the number of nuclei. All morphometric measurements were done at 100x magnification.

**Statistical analysis**

All studied parameters of the different groups are presented as mean±standard deviation. Data were analyzed using a one-way analysis of variance (ANOVA) followed by Bonferroni’s post *hoc test* or Student’s t-test, wherever applicable. All statistical analyses were done using IBM SPSS Statistics for Windows (Released 2020, Version 27.0, IBM Corp., Armonk, NY, USA). The values of p<0.05 were considered significant (Mustafa, 2020 ▶).

## Results

**Pb levels in** **blood and** **brain** **tissues**

**Figure 1:**
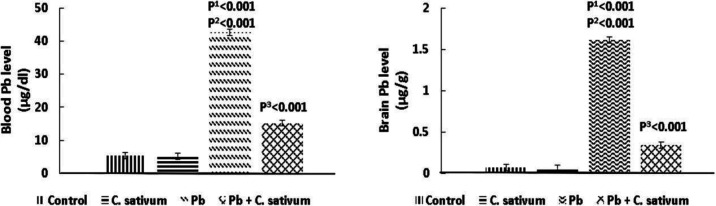
Effect of Pb and *C. sativum* co-administration on Pb concentrations (Mean±SD). p1: compared to control. p2: compared to *C. sativum*. p3: compared to Pb

There was a significant increase in the mean Pb level in the blood and cerebral cortices and cerebella in the Pb-treated groups compared to the control (p=0.001). *C. sativum* co-administration with Pb resulted in the reduction of Pb levels as compared to the control group (p=0.001) (Figure 1).

**Biochemical assays**

**Figure 2:**
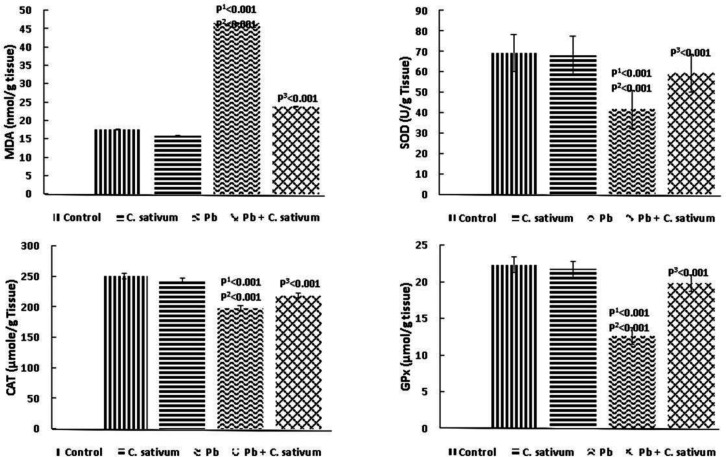
Effect of Pb and *C. sativum* co-administration on lipid peroxidation and antioxidant enzymes (Mean±SD). p1: compared to control. p2: compared to *C. sativum*. p3: compared to Pb

There was a significant increase in MDA level in the cerebral cortices and cerebella of the Pb-treated groups, which was significant as compared to the control group (p0.001). Moreover, there was a decrease in the antixodant enzymes (i.e. SOD, CAT, and GPx) levels in the Pb-treated groups, which was significant as compared to the control group (p0.001). *C. sativum* co-administration with Pb resulted in a decrease in MDA level and an increase in antioxidant enzyme (i.e. SOD, CAT, and GPx) levels (Figure 2).

**Quantitative morphometric analysis**

**Figure 3:**
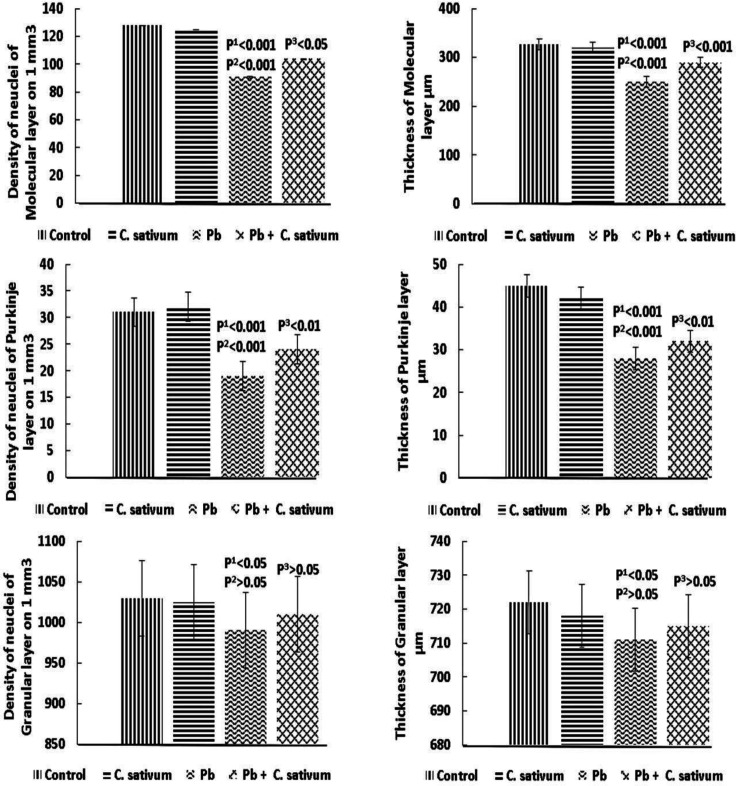
Cerebellar layers density of nuclei and thickness (Mean±SD). p1: compared to control. p2: compared to *C. sativum*. p3: compared to Pb

The cerebellum showed a normal histoarchitecture; outer molecular layer; middle Purkinje cell layer; inner granular cell layer. The molecular, Purkinje and granular nuclei density was significantly reduced in the Pb group as compared to the control (p^1^0.001 and p^2^0.001). The molecular and Purkinje nuclei density was significantly improved by administration of *C. sativum* (p0.05 and p0.01). While granular nuclei density showed no significant changes (Figure 3).

The molecular, Purkinje and granular layers thickness were significantly diminished in the Pb group as compared to control (p^1^0.001 and p^2^0.001). The molecular and Purkinje layers thickness was significantly improved by administration of *C. sativum* (p0.001 and p0.01). While granular layer thickness showed no significant changes (Figure 3).

The somatosensory cortex of the control revealed 6 layers from outside to inside which were organized as: molecular, outer granular, outer pyramidal, inner granular, inner pyramidal and polymorphic layer.

The molecular layer nuclei density significantly decreased in the Pb group as compared to the control group (p^1^0.001 and p^2^0.001) and was significantly improved by administration of *C. sativum *(p^3^0.001). The molecular layer thickness increased significantly in the Pb group as compared with the control group (p^1^0.001 and p^2^0.001) and was significantly improved by administration of *C. sativum *(p^3^0.001). The outer granular layer nuclei density and thickness significantly diminished in Pb group as compared to the control group (p^1^0.001 and p^2^0.001) and were significantly improved by treatment with *C. sativum *(p^3^0.001) (Figure 4a). 

The outer pyramidal layer nuclei density and thickness significantly diminished in the Pb group as compared to the control (p^1^0.001 and p^2^0.001) and were significantly improved by treatment with *C. sativum *(p^3^0.001). The inner granular layer nuclei density and thickness significantly diminished in the Pb group as compared to the control group (p^1^0.001 and p^2^0.001) and were significantly improved by treatment with *C. sativum *(p^3^0.001) (Figure 4b). 

The inner pyramidal layer nuclei density and thickness significantly diminished in the Pb group as compared to the control (p^1^0.001 and p^2^0.001) and significantly were improved by treatment with *C. sativum *(p^3^0.001). The polymorphic layer nuclei density and thickness significantly diminished in the Pb group as compared to the control group (p^1^0.001 and p^2^0.001) and were significantly improved by treatment with *C. sativum *(p^3^0.001) (Figure 4c). 

**Figure 4A:**
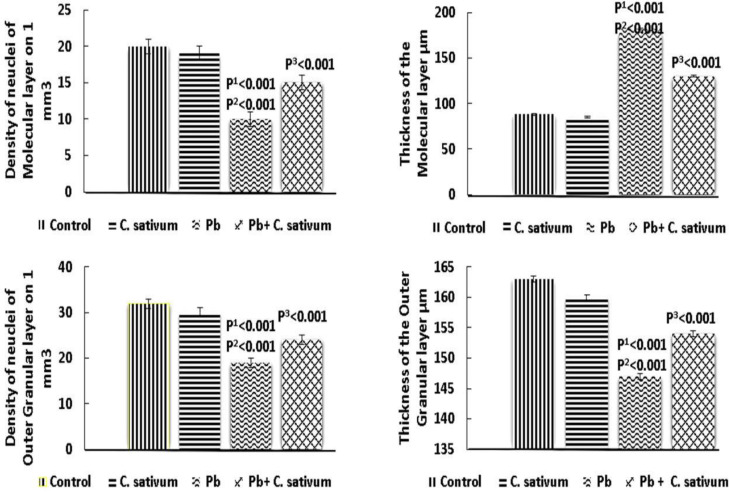
Somatosensory cortex layers density of nuclei and thickness (Mean±SD). p1: compared to control. p2: compared to *C. sativum*. p3: compared to Pb

**Figure 4B:**
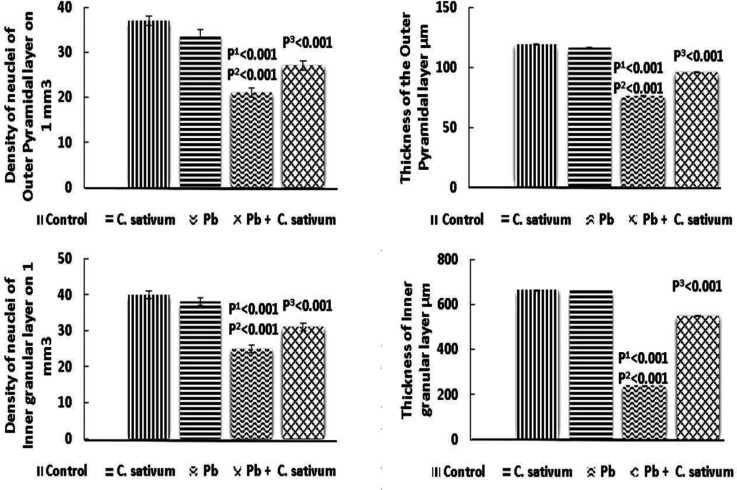
Somatosensory cortex layers density of nuclei and thickness (Mean±SD). p1: compared to control. p2: compared to *C. sativum*. p3: compared to Pb

**Figure 4C:**
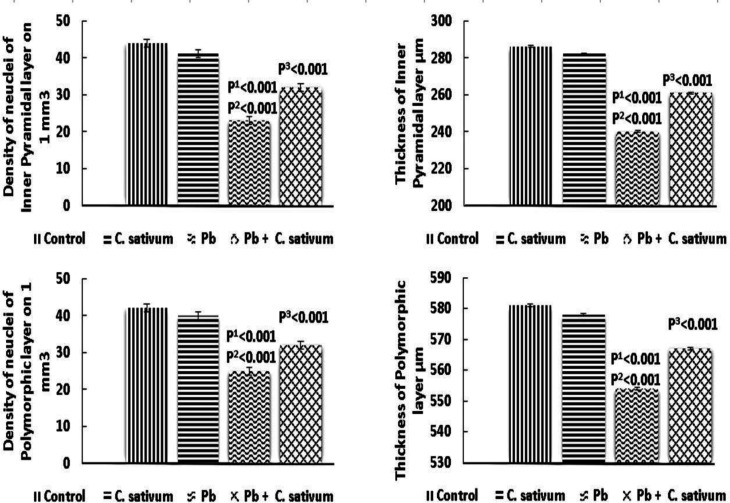
Somatosensory cortex layers density of nuclei and thickness (Mean±SD). p1: compared to control. p2: compared to *C. sativum*. p3: compared to Pb

## Discussion

The clarification of structural disorders in the nervous tissues beneath certain impacts is critical in the up-to-date neurophysiology. In spite of the accessible information on variations in the morphology of the nervous tissues subsequent to Pb actions and the role of the herbal medication, different inquiries are still in-need to be understood (Shubina et al., 2019 ▶). 

Morphometry provides a quantitative understanding of the disorder of the tissues and helps to diagnose several pathological variations at a high level of accuracy. This technique helps in the clarification of the uniqueness of response of nervous tissue to the influence of opposing stimuli on the tissues. In addition to extend the concepts about nervous system architecture (Shubina et al., 2019 ▶).

Pb toxicity causes a rise in MDA level and high oxidative stress, as MDA is considered the main product formed by lipid peroxidation subsequent to high oxidative stress which causes an excessive production of free radicals that are accountable for impaired cellular functions. Moreover, lipid peroxidation leads to irreversible damage of cell membrane, this is agreed with previous work (El-Aziz Tahoun et al., 2018 ▶). In addition, reduced concentrations of SOD, CAT and GPx activities may imitate oxidative stress in Pb group. SOD is the first-line of resistance against reactive oxygen species (ROS) and is active in catalyzing detoxification of superoxide radical. Additionally, glutathione is accounted as the main antioxidant enzyme for Pb toxicity, it was proposed that the rise of glutathione happens to compensate the free radicals formed by Pb toxicity (Saleh et al., 2019 ▶). 

Likewise, catalase is an enzymatic scavenger antioxidant, that removes cellular superoxide and peroxides and neutralize ROS before their reaction with metal catalysts to form reactive species. Additionally, it catalyzes the reduction of hydroperoxides thereby defends cells from oxidative damage (Mustafa and Hussein, 2016 ▶). The pathogenesis of brain injury is bio-activation of free radicals or involvement of a deadly agent that provokes a protein dysfunction and an immune response, depletion of reduced glutathione, oxidative stress, DNA damage and lipid peroxidation (Qadir and Ahmad, 2017 ▶).

These parameters were improved with addition of *C. sativum*, this due to the presence of flavonoids and ascorbic acid which act as antioxidants by free radical scavenging (Obafemi et al., 2019 ▶).

Morphometric analysis displayed that Pb caused a reduction in all layers of the cerebellar cortex and a significant decrease in the density of cerebellar neurons as compared with the control group (Shubina et al., 2019 ▶). These parameters were improved with co-addition of *C. sativum*. In the somatosensory cortex, results displayed a significant decrease of the total thickness of the cortex and the density of neurons in all layers of the somatosensory zone quickly declined as compared to the control group that approves the fact of increasing cell death due to lead toxicity (Shubina et al., 2019 ▶). These indicators were improved by addition of *C. sativum*.

The involvement of gamma aminobutyric acid (GABA) neurotransmission in brain activity is noticeable, and subsequently flavonoids present in *C. sativum *can act on GABAergic system in the brain. It may be assumed that these compounds might interact with GABA system and be involved in the plant’s extract-induced morphometric improvement that was reported in the current study (Ramezani et al., 2008 ▶).

Besides, it was proposed that ROS induce neuronal cell damage resulting in necrosis and apoptosis (Shin et al., 2011 ▶). The defensive properties of *C. sativum*on nervous tissues’ oxidative damages were explored and it was revealed that the plant extract prohibited lipid peroxidation (Karami et al., 2015 ▶). Conversely, the brain tissues oxidative damage is a contributing factor in neural damage and memory loss that might be prohibited by antioxidant reagents (Pourzaki et al., 2017 ▶; Seghatoleslam et al., 2016 ▶).

Suggestions propose that *C. sativum* extract has an extensive application in treating pathological situations of nervous tissues, due to its defensive properties and as a respectable medication of drug-induced nervous disorders or heavy metals toxicity attributed to antioxidant and chelation action (Ghosh et al., 2017 ▶).

The existing study has focused on the effectiveness of *C. sativum*which is considered an appetizing vegetable and a traditional medicine. It is obvious from the outcomes of the investigation that supplementation with *C. sativum*aqueous extracts protected from Pb toxic effects and oxidative stress. This work agreed with the previous reports that propose the defensive properties of *C. sativum*on Pb deposition (Tellez-Lopez et al., 2017 ▶; Velaga et al., 2014 ▶). 

Pb diminishes the thickness and the density of nuclei of the cerebellar cortex and all layers of the somatosensory cortex. Aqueous extracts of *C. sativum *produced a good significant variation in most of the evaluated parameters (oxidative stress markers, antioxidants enzymes and histological alternations) and slowed down the oxidative damage induced by Pb toxicity. Further studies are needed to evaluate its pharmacokinetics and toxicity profile to determine its clinical dose. 

## Conflicts of interest

The authors have declared that there is no conflict of interest.
